# Absence of a serum melatonin rhythm under acutely extended darkness in the horse

**DOI:** 10.1186/1740-3391-9-3

**Published:** 2011-05-10

**Authors:** Barbara A Murphy, Ann-Marie Martin, Penney Furney, Jeffrey A Elliott

**Affiliations:** 1 School of Agriculture, Food Science and Veterinary medicine, University College Dublin, Belfield, Dublin 4, Ireland; 2 Department of Psychiatry, and Center for Chronobiology, University of California, San Diego, CA 92093-0109, USA

**Keywords:** melatonin, pineal, cortisol, horse, circadian, jet lag, rhythm, extended darkness

## Abstract

**Background:**

In contrast to studies showing gradual adaptation of melatonin (MT) rhythms to an advanced photoperiod in humans and rodents, we previously demonstrated that equine MT rhythms complete a 6-h light/dark (LD) phase advance on the first post-shift day. This suggested the possibility that melatonin secretion in the horse may be more strongly light-driven as opposed to endogenously rhythmic and light entrained. The present study investigates whether equine melatonin is endogenously rhythmic in extended darkness (DD).

**Methods:**

Six healthy, young mares were maintained in a lightproof barn under an LD cycle that mimicked the ambient natural photoperiod outside. Blood samples were collected at 2-h intervals for 48 consecutive h: 24-h in LD, followed by 24-h in extended dark (DD). Serum was harvested and stored at -20°C until melatonin and cortisol were measured by commercial RIA kits.

**Results:**

Two-way repeated measures ANOVA (n = 6/time point) revealed a significant circadian time (CT) x lighting condition interaction (*p < .0001*) for melatonin with levels non-rhythmic and consistently high during DD (CT 0-24). In contrast, cortisol displayed significant clock-time variation throughout LD and DD (*p = .0009*) with no CT x light treatment interaction (*p *= .4018). Cosinor analysis confirmed a significant 24-h temporal variation for melatonin in LD *(p = .0002) *that was absent in DD *(p = .51)*, while there was an apparent circadian component in cortisol, which approached significance in LD *(p = .076)*, and was highly significant in DD *(p = .0059).*

**Conclusions:**

The present finding of no 24 h oscillation in melatonin in DD is the first evidence indicating that melatonin is not gated by a self-sustained circadian process in the horse. Melatonin is therefore not a suitable marker of circadian phase in this species. In conjunction with recent similar findings in reindeer, it appears that biosynthesis of melatonin in the pineal glands of some ungulates is strongly driven by the environmental light cycle with little input from the circadian oscillator known to reside in the SCN of the mammalian hypothalamus.

## Background

In mammals, the suprachiasmatic nucleus (SCN) of the hypothalamus drives circadian (~24 h) rhythms in a variety of behavioural and physiological processes, including the sleep-activity cycle, hormone secretion, metabolism and body temperature (for recent reviews see [1,2]). Circadian rhythms are thus controlled by an endogenous oscillator that enables organisms to anticipate rhythmic environmental changes (e.g. temperature, food availability and predation pressure) and tailor their behavioural and physiological states to the most appropriate time of solar day [3,4]. Light is the primary stimulus for synchronisation of the circadian system with the 24-h period of the earth's rotation [5]. The SCN receives photic information via the retino-hypothalamic tract and subsequently transmits timing signals to peripheral tissues throughout the body [6].

As functional timing of the neural clock cannot be directly monitored in free-moving mammals, marker rhythms that reflect SCN output are used to measure circadian phase position. The nightly rise of melatonin secretion from the pineal gland is considered one of the most stable outputs from the circadian clock [7] and is thought to represent one of the best characterized mammalian adaptations to life on a rotating planet. Melatonin is synthesized and secreted primarily during the dark period of the light/dark (LD) cycle, thereby encoding the duration of darkness and reflecting seasonal change in the length of day and night. In doing so it provides a neuroendocrine signal (from clock to body) that conveys seasonal timing and regulates reproduction in seasonal breeding animals [8,9]. Plasma levels of melatonin, cortisol, and core body temperature have historically been used as markers of circadian phase position [7,10,11]. In diurnal mammals, there exists an inverse relationship between plasma melatonin and cortisol circadian rhythms with the start of the quiescent period of cortisol production phase locked approximately to the onset of melatonin production [12]. In humans, melatonin secretion is highest during the hours of darkness, declines in the early morning and stays low during the daytime. In contrast, the 24-h pattern of plasma cortisol concentration peaks in the early morning, declines in the afternoon and remains low most of the night displaying a diurnal rhythm in humans [10] and horses [13-16] that provides temporal regulation of mammalian immune parameters through powerful immuno-suppressant activity [17].

In humans, deleterious disruption of the circadian system occurs in response to rotational shift work and transmeridian travel [18]. Rapid air travel across multiple time zones results in a disruption of synchronisation such that the previously entrained phase timing of the biological clock leads to temporal conflict with the new cycle of light and dark (LD). This phenomenon is known as jet lag, and is characterised by fatigue, disturbed sleep, depression, gastrointestinal disturbance, reduced cognitive capacity and physical performance deficits [19-22]. These symptoms, which can persist for days until the circadian system adjusts to the new environmental conditions, are of particular concern for athletes competing at international destinations. An initial investigation into the severity and longevity of jet lag in the equine athlete examined re-entrainment rates of plasma melatonin and core body temperature following an abrupt 6-h phase advance of the LD cycle [23]. In contrast to studies that demonstrate a gradual adaptation of melatonin rhythms to an advanced photoperiod [24-28], we found instead that equine melatonin rhythms were re-entrained to a 6-h LD phase advance on the first post-shift day. This surprising result led to the present study.

A 24-h rhythm can only be defined as circadian when it persists in constant conditions, such as constant darkness (DD), or constant light (LL). This continuance of ~24 h oscillations in a physiological or behavioural variable under constant conditions indicates that the observed rhythm is endogenously controlled, and not merely a driven response to environmental time cues. Although the 24-h rhythm of equine core body temperature has demonstrated robust circadian regulation under LL [29], the circadian rhythms of melatonin and cortisol in the horse have not previously been examined under constant conditions. The aim of the current study was to determine the temporal pattern of melatonin and cortisol in the horse under the constant lighting condition of extended darkness (DD). Establishing the expected circadian regulation of these hormones would validate their continued use as physiologically relevant markers of circadian phase in future studies investigating the effects of jet lag on equine athletes.

## Methods

All animal procedures were approved by the University College Dublin Animal Research Ethics Committee. Six healthy mares aged between 5 and 11 years and of mixed light horse breed were used in this study. Mares were maintained outdoors under natural photoperiod for one month prior to the experiment (longitude W6.8, latitude N53.2, County Kildare, Ireland), which was conducted at a time of year (Sept 2^nd ^- Sept 4^th^, 2008) corresponding approximately to a 13.5 h light and 10.5 h dark (LD 13.5:10.5) artificial light cycle. Barn lighting reflected the timing of ambient dawn and dusk with lights on at 06:38 h and lights off at 20:08 h. The light intensity in the barn was measured using a LUXmeter (LX-1010 B Digital Lux Meter) as 200 - 250 Lux at the horses' eye level. The day before initiation of sampling mares were housed in individual stalls in a lightproof barn (DD = < .5 Lux) and the left jugular furrow of each mare was clipped and surgically prepared for placement of indwelling jugular catheters (MILA International, Florence, KY). The jugular catheter was secured in place with suture (3 metric Monosof^® ^nylon, Gosport, UK) and bandage. Blood samples were collected for 24 h while mares remained under the LD cycle and, without turning lights on the following morning, for a further 22 h under constant darkness (DD). Blood sampling commenced at 07:00 h, here designated Zeitgeber Time (ZT) 0 and continued at 2-h intervals, first for 24-h under LD (ZT 0 - ZT 24), and then for 24-h of extended dark under DD (CT 0 - CT 24), with the last sample at 05:00 h (CT 22) of the second sampling day (where ZT 24 = CT 0). Hay and water were provided *ad libitum *throughout the trial and were topped up at 4-h intervals to avoid a conspicuous 24-h temporal cue [29]. Temperature inside the barn remained relatively constant for the duration of the trial, ranging from 16-18°C. Blood samples (6 ml; n = 6 per time point) were collected into heparinized blood tubes (BD Vacutainer Systems, Plymouth, UK). Patency of catheters was maintained using heparinized saline flush. Blood samples were stored at ambient temperature for 2 h and kept overnight at 4°C. The next day, samples were centrifuged at 1600 × g for 20 min, serum was decanted and was immediately stored at -20°C until subsequent analysis. Samples were collected throughout the hours of darkness with the aid of dim red flashlights (< 5 Lux). At each time point, samples were collected from the mares in the same order (requiring ~15 min), and care was taken to ensure mares were only minimally disturbed by the procedure.

### Melatonin radioimmunoassay (RIA)

Melatonin was measured using a Bühlmann melatonin RIA kit (RK-MEL2, ALPCO Diagnostics, Windham, NH). Serum aliquots (500 μl) were column extracted according to the directions of the manufacturer and reconstituted in 500 μl of incubation buffer solution provided with the kit. Aliquots of the reconstituted extracted samples (200 μl) were assayed in duplicate in a single assay which also included kit low and high controls that confirmed assay performance (averaging 2.28 and 17.63 pg/ml respectively). As documented by the manufacturer, the efficiency of the extraction method is > 90%, while the assay has an estimated functional sensitivity (CV = 10%) of 0.9 pg/ml and an estimated analytical sensitivity of 0.3 pg/ml. This assay has been used previously to examine MT levels in equine serum [23].

### Cortisol radioimmunoassay (RIA)

Cortisol was measured using a Coat a Count assay kit (Siemens, LA, USA). 25 μl of serum samples, QC samples (at three levels) and calibration standards (0-50 μg/dl) were aliquoted in duplicate into cortisol antibody coated tubes. 1.0 mL of 125I cortisol tracer was added to each tube and the tubes were incubated in a waterbath at 37°C for 45 minutes. After this time, the tubes were decanted thoroughly and counted using the Wizard 1470 gamma counter (Perkin Elmer/Wallac, Turku, Finland). The sensitivity of the assay was 0.2 μg/dl. The CV% for the Quality Control samples at low, medium and high levels were 16.5, 10.9 and 8.1%, respectively.

### Data Analysis

**Figure 1:**
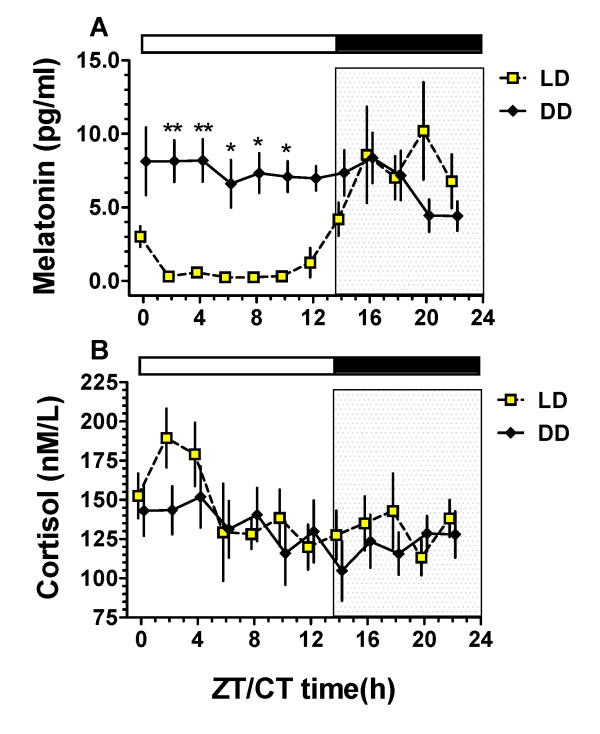
**(A-B): Averaged equine MT (A) and cortisol (B) rhythms under conditions of light dark (LD 13.5:10.5) and constant darkness (DD)**. The barn LD cycle is depicted above each graph: white bars represent light in LD and subjective day in DD; black bars and internal shading represent darkness in LD and subjective night in DD (CT14-24). Sampling began at ZT/CT0 in LD and ended at CT22 in DD after 32 h in continuous darkness. Hormone data are presented as mean ± SE for six mares (n = 6). CT0 represents 0700 h; CT2 0900 h, etc. (A) MT remained low during hours of light (L) in LD but not during the corresponding times (subjective day, CT2-CT10) in DD. A 24-h MT rhythm is evident under LD conditions, but not under DD (*p *< 0.0001). *, ** denote significant difference (*p *< .05, *p *< .01) at specific time points (Bonferoni post hoc tests). (B) In contrast, cortisol showed similar 24-h patterns in LD and DD.

Two-way repeated measures analysis of variance (ANOVA) (LD/DD cycle × Time) was used to assess differences in MT and cortisol between 24-h LD and DD sample collections. Bonferroni post-hoc tests were used to evaluate differences between time points where appropriate. Data was analyzed using GraphPad Prism Version 4.0 for Windows (GraphPad Software, San Diego, CA), and are presented as time point means ± SE (Figure 1). A value of *p < .05 *was considered significant. The presence of circadian (24-h) temporal variation for the group means was evaluated using the Cosinor programme of Refinetti et al (2007) [30] based on the least squares cosine fit method of Halberg et al (1967) [31] and also by separately computing cosine fits to the hormone values for each mare over the first 24 h (LD) and the final 24 h (DD) (n = 12 data points/series).

## Results

**Figure 2:**
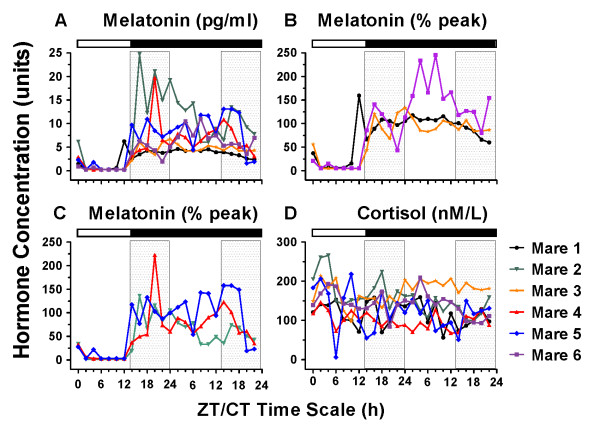
**(A-D): Individual equine MT (A-C) and cortisol (D) time series throughout the experimental LD and constant dark (DD) conditions described for Figure 1**. Due to substantial individual differences in peak MT levels expressed in the first hours of darkness individual MT data were normalized and expressed as a percentage of the ZT16-ZT22 mean (set to 100%). The resulting plots (B, C) illustrate the two different temporal patterns discussed in the text: continuously high levels in B contrasting with eventual MT declines in C. Panel D illustrates the substantial individual and ultradian variation in blood cortisol. Other conventions are the same as in Figure 1.

Two-way repeated measures ANOVA (n = 6/time point) of hormone levels revealed a significant circadian time (ZT/CT) x light treatment interaction (*p < .0001*) for melatonin with mean levels remaining consistently high during DD, and thus elevated relative to LD throughout the subjective day (i.e. at CT 2,4,6,8 and 10, Figure 1A). In contrast there was no difference between the cortisol profiles in LD and DD however, a significant variation over time was observed (*p = .0009*) (Figure 1B). Observing substantial individual differences in the amplitude and pattern of temporal variation of melatonin in horse serum (pg/ml) (Figure 2A), we normalized the individual data by expressing the value at each time point as a percentage of the ZT16-ZT22 mean, an elevation representing the nocturnal LD peak (i.e. peak average set to 100%). Viewing the data in this way (Figure 2B-C) revealed two distinguishable patterns. In 3 mares (Figure 2B), MT rose rapidly between ZT12-ZT16, thereafter remaining elevated, but with notable fluctuations in Mare # 6. In the other 3 mares (Figure 2C), the initial evening rise was followed eventually by a notable decline, either between ZT22 and CT8 (Mare #2), or not until the last few hours (h 28-32) of extended dark at subjective circadian times CT16-CT22 (Mares # 4,5).

Cosinor analysis of group mean data confirmed a significant circadian component for melatonin in LD *(p = .0002) *that was absent in DD *(p = .51)*. In contrast, by cosine analysis of group mean values, cortisol was clearly circadian in DD *(p = .0059) *but the 24-h cosine fit was shy of significance in LD *(p = .076) *(Table 1). The *p *values for cosine fits to the LD and DD time series (12 points each) of each individual mare are also reported in Table 1 while corresponding raw data curves appear in Figure 2D.

**Table 1:** Significance (*p*) values from 24-hour Cosine fits to melatonin (MEL) and cortisol (Cort) time series for individual mares during LD and DD and for corresponding group means (12 points/fit).

24-h fits	Mel LD	Mel DD	Cort LD	Cort DD
Mare 1	.013	.01	.6	.07

Mare 2	.0038	.1	.21	.37

Mare 3	.002	.83	.2875	.2858

Mare 4	.036	.07	.7436	.7473

Mare 5	.0016	.22	.53	.11

Mare 6	.0015	.016	.2	.002

**Group Mean (n = 6)**	**.00026**	**.51**	**.076**	**.005**

## Discussion

In accordance with previous studies, [16,23,32]our results demonstrate a robust 24-h rhythm in equine plasma MT values under an LD cycle. Surprisingly, this rhythmicity disappeared when mares were maintained in extended darkness (DD), providing no direct evidence for circadian regulation of this important internal temporal cue in the horse. Specifically, following normally scheduled lights out in the barn (~ ZT 13.5) mean melatonin levels rose rapidly, achieving expected night time values within 2.5 h (ZT 16). Thereafter, through 32 h of extended darkness (DD), mean MT values remained high. Furthermore, throughout this exposure to extended dark, no individual mare showed either the expected decline in serum MT beginning 10-12 h into continuous dark (e.g. around expected dawn at CT 0), or a subsequent rise coincident with subjective dusk (~ CT 14) after 24 h in continuous dark. These findings suggest that under natural conditions, melatonin inhibition in the horse occurs in response to light and not through an endogenous mechanism. It is worth noting that a non-significant decline in group mean MT levels appeared in the last 6 h of darkness (CT 18-22) as a result of a reduction in serum MT values for 3 of the 6 mares. A plausible explanation could be exhaustion of pineal synthetic activity after some 28-30 h of continuous activity in extended darkness. Alternatively, these observations could be interpreted as consistent with the emergence of an initially masked circadian signal that could have potentially become more apparent had the duration under DD not been limited to 32 h. However, this would imply that the underlying circadian period differs widely from 24 h, as the observed decline in MT began late, at about CT6 in one mare, and at about CT 14 to CT 20 in the other two. This interpretation, which postulates extremely variable circadian periods in DD, is also not supported by the cortisol data presented here, or by previously reported ~ 24 h circadian rhythms in activity and gene expression observed in these same horses under identical conditions [33].

Thus, for melatonin, the straight forward interpretation is that under 32 h of continuous darkness (DD), neither group mean nor individual MT values demonstrate circadian regulation. In contrast, mean cortisol levels showed 24-h rhythmicity in both LD and DD, with group cosinor analysis demonstrating a more robust circadian (24 h) component in DD. The greater strength of the 24-h variation in DD compared to LD is also evidenced by higher *p *values for individual cosine fits in four of the mares in DD compared to LD. The relatively high variance in the cortisol time point means throughout LD and DD (Figure 1B) and irregular ups and downs in the individual profiles (Figure 2D) may relate to studies showing that minor perturbations in the environment can eliminate the cortisol rhythm in horses [34]. Thus visual stimulation during the photophase combined with human activity throughout LD and DD may have increased arousal at sampling and feeding times, thereby resulting in increases in the overall level and variation in cortisol secretion.

Previously, plasma MT was measured in four mares at different times of the year, namely the summer and winter solstices, and the spring and autumn equinoxes [32]. Blood samples were collected for 24 h from mares individually housed under natural photoperiod conditions, and for a further 24 h from mares exposed to acutely extended darkness (total darkness for 3-4 h before and after the natural sunrise and sunset). The authors reported a 24-h rhythm in MT secretion at each season under an LD cycle, whereby MT elevation corresponded to the night length. Accordingly, the mean duration of elevated MT varied at each season, with the longest duration observed in winter and the shortest observed in summer. In extended darkness, the elevations in MT were higher than those measured under natural photoperiod at all seasons except summer. Furthermore, the rise and fall of nocturnal MT elevations occurred before and after comparable onset/offset times measured under natural photoperiod, instead mirroring the acutely extended darkness of the artificial LD cycle to which the animals were exposed. Observing this phenomenon at each season led the authors to suggest that in horses, natural environmental light, both at dawn and dusk, gates the full expression of the SCN neural signal. The SCN, in turn, regulates the daily pattern of MT secretion [32]. However, an alternative explanation supported by the data presented here is that equine daily MT rhythms are directly driven by the environmental photoperiod, rather than via circadian pacemaker control. These ideas appear consistent with the observed immediate resynchronization of the MT rhythm in horses following an abrupt 6 h advance of the LD cycle [23]. Additionally, the rapid 6-h phase advance of MT that we observed contrasts starkly with previous observations of a gradual advance in melatonin onset when human subjects are exposed to a comparable 6 h phase advance of the LD cycle [26,35] and highlights the need for improved understanding of species differences in the photic and circadian regulation of melatonin synthesis and secretion.

The surprising absence of a circadian MT rhythm in DD is consistent with the conclusion that there is no endogenous circadian regulation of MT synthesis in the horse, at least under classic free-running conditions of continuous darkness. Data opposing this conclusion have been demonstrated in hamsters, primates and sheep [36-38] where levels of MT were shown to rise spontaneously during subjective nights. Ours is clearly an unexpected finding but the simultaneous demonstration here of a cortisol circadian rhythm in DD, and previously, that in constant conditions horses display other circadian rhythms, including those in body temperature [27], peripheral clock gene expression and locomotor activity [33] implies that horses, like other vertebrates, possess a fully competent, self sustained circadian pacemaker presumably in their SCN. Thus, the intriguing questions are: How and why is it that the equine melatonin rhythm fails to persist as a circadian rhythm in continuous darkness? Has MT synthesis become totally uncoupled from circadian (SCN) regulation? Alternatively, are the circadian oscillators regulating melatonin secretion in the horse highly dampened? Whatever the mechanism, is circadian regulation absent at all times, or is the absence restricted to a particular season, or set of experimental conditions? Such questions can only be answered by further study.

It is worth highlighting the known differences in regulation of melatonin production between rodents and ungulates. In contrast to rodents, the nocturnal rise of melatonin arylalkylamine N-acetyltransferase (AA-NAT) in sheep is not accompanied by a similar rise in AA-NAT mRNA expression [39], such that the biosynthesis of MT is primarily gated by post-translational control [40]. Johnston et al (2004) have extended these findings of interspecies differences by demonstrating that the ovine pineal also differs from that of the rodent by the absence of rhythmic expression of inducible cyclic AMP early repressor (ICER) and Cryptochrome 1 and the authors suggest that this may reflect evidence of differences in evolutionary divergence between ruminants and rodents [41]. It is possible that as yet unrevealed differences in melatonin regulation may exist between ruminants and non-ruminant ungulates such as the horse, that in turn reflect the evolutionary timeline since the phylogenetic split between Artiodactyls and Perissodactyls and the emergence of differences in their adaptive lifestyles.

A lack of rhythmicity in production of the MT hormone has also been observed in reindeer [42]. Similar to the results obtained in the current study, reindeer displayed robust MT rhythmicity when housed under an LD cycle. However when animals were placed in DD for 72 h, their MT levels increased and remained significantly higher than daytime levels for about 24 h, fell to baseline for about 12 h, and then rose again expressing a second ~ 24 h elevation, thus oscillating in DD (with a period of about 36 h) but also failing to demonstrate a ~24-h circadian rhythm. Stokkan et al. (2007) postulate that to maintain precise seasonal timing in an extreme environment, MT secretion in reindeer, and also perhaps in other Arctic animals, is driven directly by changes in photoperiod and not by circadian machinery [42]. A more recent study in reindeer showed acute daytime elevations of melatonin in short (2.5 h) intervals of darkness experienced during the ambient photophase (in a single 2.5D:2.5L:2.5D cycle) and that, in contrast to rat [43], cultured fibroblast cells from this species do not exhibit robust circadian rhythms in clock gene expression [44]. In this regard, horses are less exceptional as robust clock gene rhythmicity has been demonstrated both in cultured fibroblasts and in peripheral tissue biopsies [33,45]. Reindeer are similar to horses in that they are large seasonal breeding (albeit short-day breeding) ungulates. Lu et al (2010) speculate that entrainment of annual reproductive cycles in reindeer may depend on informative melatonin signals confined to specific times of year. Similarly in the horse, it is appealing to consider whether the amplitude of the MT rhythm, the strength of its photoperiodic/SCN regulation, or the ability to display a circadian rhythm in continuous darkness, may vary with season or time of year. These are questions ripe for future study. In particular, it will be interesting to investigate the pattern of MT secretion under continuous dim illumination (of an intensity equivalent to natural starlight and/or moonlight) at different seasons, particularly in advance of the onset of the mare's natural breeding season (April - May).

## Conclusions

This study has revealed the unexpected failure of the daily rhythm of equine MT to persist as a circadian rhythm in DD, implying that MT is not a suitable marker of circadian phase in horses. In contrast, the less robust daily rhythm in cortisol persisted as a circadian rhythm in DD. Additionally, the present findings alter the implications of a jet lag study in which we reported rapid re-entrainment of the equine MT rhythm following a 6-h phase advance of the LD cycle. That is, the present finding of an absence of circadian variation in MT in continuous darkness, suggests that this rapid realignment of the MT rhythm should instead be viewed as further evidence that in the horse the MT rhythm is largely driven by the LD cycle, rather than entrained by it in circadian fashion. Because the 24 h melatonin rhythm is a recognized internal temporal signal in mammals, and is able to contribute to resetting and entrainment of the SCN clock [27,46], it's rapid adjustment to the external LD cycle may have important consequences for the broader temporal adjustment of the equine circadian system following transmeridian travel, even though MT itself, may not qualify as a good marker of SCN phase. Further, the present results provide impetus for new studies to identify additional robust markers of circadian phase in the horse so that we may better understand the effects of transmeridian travel on temporal aspects of equine physiology and behaviour.

## Competing interests

The authors declare that they have no competing interests.

## Authors' contributions

BAM conceived of the study and coordinated the study design, sample collection, data analysis and interpretation, and prepared the manuscript. JAE contributed to study design, data analysis, interpretation and figure preparation, ran the MT RIA, and helped prepare the manuscript. AMM contributed to study design, sample collection, data analysis and preparation of the manuscript. PF conducted the cortisol RIA and contributed to manuscript preparation. All authors read and approved the final manuscript.
